# Hemodynamic force analysis is not ready for clinical trials on HFpEF

**DOI:** 10.1038/s41598-022-08023-4

**Published:** 2022-03-07

**Authors:** Per M. Arvidsson, Anders Nelsson, Martin Magnusson, J. Gustav Smith, Marcus Carlsson, Håkan Arheden

**Affiliations:** 1 Clinical Physiology, Department of Clinical Sciences Lund, Skåne University Hospital, Lund University, 22185 Lund, Sweden; 2 Department of Cardiology, Clinical Sciences, Skåne University Hospital, Lund University, Malmö, Sweden; 3 Department of Cardiology, Clinical Sciences, Skåne University Hospital, Lund University, Lund, Sweden; 4 Department of Clinical Sciences Malmö, Lund University, Malmö, Sweden; 5 Wallenberg Center for Molecular Medicine, Lund University, Lund, Sweden

**Keywords:** Heart failure, Diagnostic markers

## Abstract

Hemodynamic force analysis has been proposed as a novel tool for early detection of subclinical systolic dysfunction in heart failure with preserved ejection fraction (HFpEF). Here we investigated the ability of hemodynamic forces to discriminate between healthy subjects and heart failure patients with varying degrees of systolic dysfunction. We studied 34 controls, 16 HFpEF patients, and 25 heart failure patients with mid-range (HFmrEF) or reduced ejection fraction (HFrEF) using cardiac magnetic resonance with acquisition of cine images and 4D flow at 1.5 T. The Navier–Stokes equation was used to compute global left ventricular hemodynamic forces over the entire cardiac cycle. Forces were analyzed for systole, diastole, and the entire heartbeat, with and without normalization to left ventricular volume. Volume-normalized hemodynamic forces demonstrated significant positive correlation with EF (r^2^ = 0.47, p < 0.0001) and were found significantly lower in heart failure with reduced ejection fraction compared to controls (p < 0.0001 for systole and diastole). No difference was seen between controls and HFpEF (p > 0.34). Non-normalized forces displayed no differences between controls and HFpEF (p > 0.24 for all analyses) and did not correlate with EF (p = 0.36). Left ventricular hemodynamic force analysis, whether indexed to LV volumes or not, is not ready for clinical trials on HFpEF assessment.

## Introduction

Hemodynamic forces, essentially the exchange of forces between intracardiac blood and surrounding myocardium, have garnered attention as potential quantitative markers of cardiac health. Hemodynamic forces indicate the functional performance of the ventricle, and have been proposed as a new, noninvasive measure of ventricular function as well as a possible driving mechanism for cardiac remodeling^1^. Hemodynamic forces were initially studied in heart failure patients with dyssynchronous left ventricles, where certain force patterns have been suggested to convey prognostic information regarding the response to cardiac resynchronization therapy (CRT)^1–3^. Early studies used echocardiographic particle imaging velocimetry (Echo-PIV) to compute hemodynamic forces in the apical three-chamber view, resulting in a limited approximation of the total force field. Later developments saw the application of cardiovascular magnetic resonance 4D flow to compute three-dimensional hemodynamic forces for the entire left ventricle (LV) and right ventricle (RV) over the cardiac cycle, a method now considered to be the reference standard^4,5^. Employing this reference method, several studies uncovered new mechanistic information regarding the rerouting of blood flow through both ventricles in both health and disease^6–8^.

In parallel to the more direct flow-based methods, a numerical framework for approximation of hemodynamic forces from regular long-axis steady-state free precession (SSFP) images has been developed^9^ and validated^10^. While not as precise or accurate as the 4D flow-based approach, the numerical method could potentially be retrospectively applied to large cohorts of clinical data, to search for prognostic markers in various stages of structural heart disease even when 4D flow data has not been acquired. In a promising recent study, Lapinskas et al. used the numerical method to compute LV hemodynamic forces in healthy controls and heart failure with preserved ejection fraction (HFpEF) patients^11^. The authors found decreased longitudinal (apical-to-basal) hemodynamic forces in HFpEF patients despite having unaltered longitudinal and circumferential strain, suggesting a potential for early detection of subclinical myocardial systolic dysfunction, as well as enabling evaluation of therapeutic response in this patient group. To date, no studies have used reference-standard 4D flow measurements to evaluate hemodynamic forces in HFpEF.

The aim of the current study was therefore to quantify hemodynamic forces in patients with heart failure with varying degrees of systolic dysfunction using the best available measurement technique, testing the hypothesis that hemodynamic forces are quantitatively altered in HFpEF patients compared to both healthy controls and heart failure patients with reduced ejection fraction.

## Methods

### Study design

This cross-sectional study was performed at Skåne University Hospital (Lund, Sweden). All participants provided written consent after receiving written and oral information about the study. Study participants were divided into two groups: healthy controls and heart failure patients.

Healthy controls were recruited by invitation after participating in the Malmö cohort of the Swedish CArdioPulmonary bioImage Study (SCAPIS)^12^, which is a contemporary, prospective population-based cohort study including echocardiography and coronary computed tomography (CT) angiography image acquisition at the baseline examination^12^. Controls were defined as having no history of heart failure symptoms, normal echocardiogram and electrocardiogram (ECG), no signs of coronary vessel disease on a cardiac CT scan, blood pressure ≤ 140/90 mmHg, no systemic disease, no lung disease, and no medications.

Heart failure patients were recruited from the Cardiology Clinic, Skåne University Hospital, after being diagnosed by a cardiologist. The assessment included patient history of heart failure symptoms, clinical examination, and lab workup. Patients were subsequently classified according to LV EF as measured from CMR images. Heart failure with preserved ejection fraction was defined as EF ≥ 50%, HFmrEF as EF below 50 but ≥ 40%, and HFrEF as having EF < 40%.

Exclusion criteria were atrial fibrillation, more than mild aortic regurgitation or stenosis, inability to undergo CMR exam (claustrophobia, inability to lie in the supine position, known hypersensitivity to gadolinium contrast agent, metal fragments in body, devices), and known systemic disease apart from heart failure. Patients with mitral insufficiency were not excluded as this is a common secondary manifestation of heart failure with LV dilatation.

### Magnetic resonance imaging

All subjects underwent imaging at 1.5 T using a Magnetom Aera or Sola (Siemens Healthcare, Erlangen, Germany). The scan protocol included balanced steady-state free precession (bSSFP) cine images in the 2-, 3-, 4-chamber and short axis views, through-plane flow measurement in the proximal aorta, first-pass perfusion imaging during adenosine stress, postcontrast 4D flow sampled from a box covering the entire heart and proximal great vessels, and late gadolinium enhancement (LGE) imaging for assessment of myocardial viability. All images were acquired using retrospective ECG gating.

4D flow was acquired using a previously validated gradient recalled echo sequence (WIP 785 K) with Cartesian readout^13,14^. Scanning was performed with a respiratory navigator prepulse centered on the right liver lobe in 9 subjects, and without navigator in 66 subjects to decrease scan time. A previous study found that quantification of hemodynamic forces was not sensitive to the use of respiratory gating^14^. Typical imaging parameters for the 4D flow: TE/TR 3.5/5.7 ms, flip angle 8°, VENC 100 cm/s, acquired temporal resolution 46 ms, 40 reconstructed time phases, spatial resolution 3 mm isotropic, no slice gap, partial Fourier factor 0.75 in phase and slice directions, no slice oversampling, GRAPPA factor 4 (factor 2 in anterior–posterior phase encode direction and 2 in superior-inferior slice encode direction) and temporal segmentation factor 2. 4D scan times were not systematically measured but were typically in the 6–10-min range, primarily dependent on cardiac dimensions, heart rate, and when used, respiratory gating efficiency.

### Data analysis

Image analysis was performed in Segment 2.3 R8408 (Medviso, Lund, Sweden) using freely available custom plugins^14,15^. Residual background phase errors were corrected by subtracting a first- or second-order polynomial fit of static tissue velocities^16^, where correction order was selected to provide the best overall background nulling near the heart in the three phase encoding directions. Phase wraps were unwrapped automatically^17^. Spatial alignment between bSSFP and 4D flow images was evaluated visually and manually adjusted when necessary.

Late gadolinium enhancement images were used to stratify patients according to the presence of ischemic scar and/or non-ischemic fibrosis and to determine heart failure etiology (ischemic or nonischemic). Images were classified as ischemic LGE, non-ischemic LGE, or negative. Ischemic LGE was defined as subendocardial or transmural areas with increased intensity and within a vessel territory. Non-ischemic LGE was defined as subepicardial or mid-mural areas of increased intensity, or transmural areas in areas where bSSFP images demonstrated normal regional function, ruling out infarction. Slight fibrosis in the right ventricular septal insertion points was considered a nonspecific finding and was classified as LGE negative.

### Computation and normalization of hemodynamic forces

**Figure 1:**
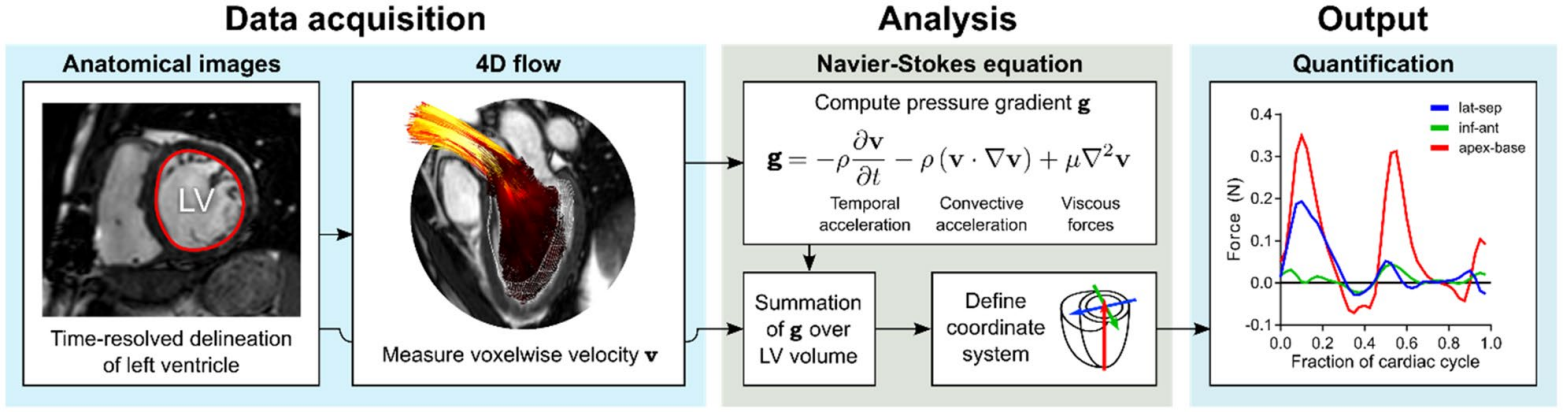
Method overview. LV segmentation is performed in standard short-axis bSSFP images and used as boundaries for the 4D flow data. The flow field is then used as input to the Navier–Stokes equation to compute the instantaneous pressure gradient, which is subsequently integrated over the LV to produce the global hemodynamic force vector. This vector can then be decomposed into three orthogonal components.

Global hemodynamic forces were computed as previously described^14^. Figure 1 provides an overview of the steps taken. In short, LV endocardial contours were delineated in the short-axis bSSFP stack across all timeframes. LV contours were then translated onto the 4D flow stack and the Navier–Stokes equation used to compute the global pressure gradient **g** within the LV for each timeframe:

$$\mathbf{g}=-\rho \frac{\delta \mathbf{v}}{\delta t} -\rho \left(\mathbf{v} \cdot \nabla \mathbf{v}\right)+\boldsymbol{ }\mu {\nabla }^{2}\mathbf{v},$$

where **v** is the velocity measured using 4D flow. Blood density was set to ρ = 1.05 g/cm^2^ and viscosity was set to µ = 4 × 10^–3^ Ns/m^2^. The instantaneous hemodynamic force was computed by integration of **g** over the LV.

Forces were decomposed into three orthogonal components using eight atrioventricular valve (AV) plane reference landmarks, as previously described^8^. The apical-basal direction was defined as being perpendicular to the AV plane. The anteroseptal-inferolateral direction was orthogonal to the apical-basal direction and oriented to intersect the mitral annulus and LV outflow tract; this plane aligns with the Society for Cardiovascular Magnetic Resonance (SCMR) recommendations for 3-chamber view orientation^18^.

Lapinskas et al. suggested normalizing forces to ventricular volume, resulting in a volume-related force index^11^. We also computed volume-normalized forces by dividing the force magnitude with LV volume for each timeframe, to facilitate comparison with previous results. Root mean square (RMS) force was computed for systole and diastole separately as well as for the entire cardiac cycle, in the transverse plane and the apical-to-basal direction. Average forces (F_RMS_) and peak forces (F_peak_) were quantified for the entire cardiac cycle and for systole/diastole separately, with and without volume normalization.

To facilitate comparison between subjects independent of heart rate, hemodynamic forces were resampled to a common time axis with end systole set to 0.42, the average in the study population. End systole was determined individually by extrapolation of the downslope of the aortic flow curve^19^.

### Statistical methods

Statistical analysis was conducted using GraphPad Prism 9.1 (GraphPad Software, La Jolla, CA) and significance was assigned at p < 0.05 for all analyses. Continuous variables are expressed as average ± 1SD unless otherwise noted.

We used the Kruskal–Wallis test to investigate differences in the main outcome measures F_RMS_ and F_peak_ between groups, as well as for population characteristics, and post hoc analysis using Dunn’s multiple-comparisons test where appropriate. Sex differences between controls and patients was evaluated using Fisher’s exact test.

### Ethics approval and consent to participate

This study was conducted in accordance with the Helsinki declaration and was approved by the Ethical Review Board, Lund, Sweden (application numbers 741/2004, 2013/891, 2013/900). All subjects provided written informed consent.

## Results

Imaging was performed on 96 subjects, of which 21 were excluded from the study after initial analysis: 8 due to poor image quality or missing data, 6 due to aortic regurgitation, 3 due to other systemic diseases, 3 patients with impaired systolic function who did not fulfill criteria for heart failure diagnosis, and one due to severe aortic stenosis.

We included 75 subjects in the final analysis. Subject characteristics are summarized in Table 1. Controls and patient groups did not differ with regard to sex, body surface area, blood pressure, resting heart rate, or cardiac index (p > 0.05 for all). Notably, the aggregated patient group was older than controls, driven by HFpEF patients (70 vs 62 years, p = 0.01).

**Table 1:** Subject characteristics.

	Controls	HFpEF	HFmrEF	HFrEF	p value	Dunn’s multiple comparisons test
Number of subjects	34	16	9	16		
Age, year	62	70	64	65	**0.02**	0.01 for controls vs. HFpEF
Sex	17m, 17f	9m, 7f	5m, 4f	12m, 4f	0.35*	
Body surface area, m^2^	1.9	2.0	2.0	2.0	0.66	
Systolic blood pressure, mmHg	127	128	124	128	0.91	
Diastolic blood pressure, mmHg	78	70	70	75	0.13	
Stroke volume, ml	94	101	99	74	**0.02**	0.02 for HFpEF vs. HFrEF
Heart rate, beats/min	64	59	64	69	0.06	
Cardiac index, l/min/m^2^	3.0	3.0	2.9	2.7	0.16	
End-diastolic volume, ml	160	174	205	281	**< 0.0001**	> 0.999 for HFpEF vs controls, < 0.05 for HFmrEF vs controls, < 0.0001 for HFrEF vs controls
Ejection fraction, %	58	59	47	29	0.54^†^	
LGE pattern		3 ischemic, 2 nonischemic, 11 negative	1 ischemic, 2 nonischemic, 6 negative	4 ischemic, 8 nonischemic, 3 negative, 1 missing^‡^		
HF etiology		3 ischemic, 13 nonischemic	0 ischemic, 9 nonischemic	5 ischemic^‡^, 11 nonischemic		

Significant values are in bold.

Average values.

*Comparison between controls and aggregated patients, computed using Fisher’s exact test.

^†^Comparison between controls and HFpEF, computed using two-tailed Student’s T test.

^‡^LGE images were not available in one patient; this case was classified as ischemic due to dilated ventricle with regional hypo/akinesia in distal parts of the LAD territory with no global hypokinesia.

**Figure 2:**
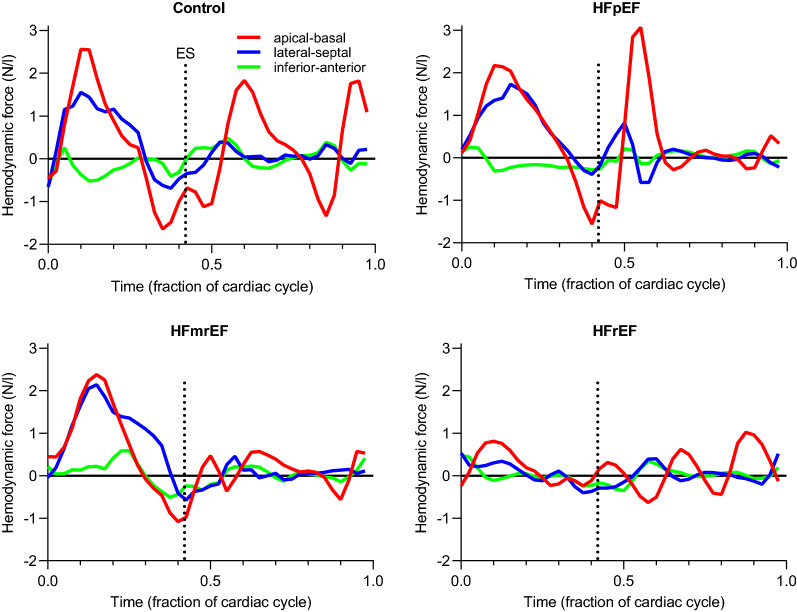
Examples of volume-normalized hemodynamic force patterns in the four groups. The vertical dotted line denotes end systole (ES).

Examples of volume-normalized hemodynamic forces are shown in Fig. 2. The force patterns in the control and the HFpEF patient displayed slight differences in the apical-basal direction, particularly during diastole (panels A and B). In the HFmrEF patient, lower forces were seen in the apical-basal direction during diastole (panel C). In the HFrEF patient with moderately dilated LV (end-diastolic volume 273 mL, EF 20%), volume-normalized forces were lower in both systole and diastole (panel D).

**Figure 3:**
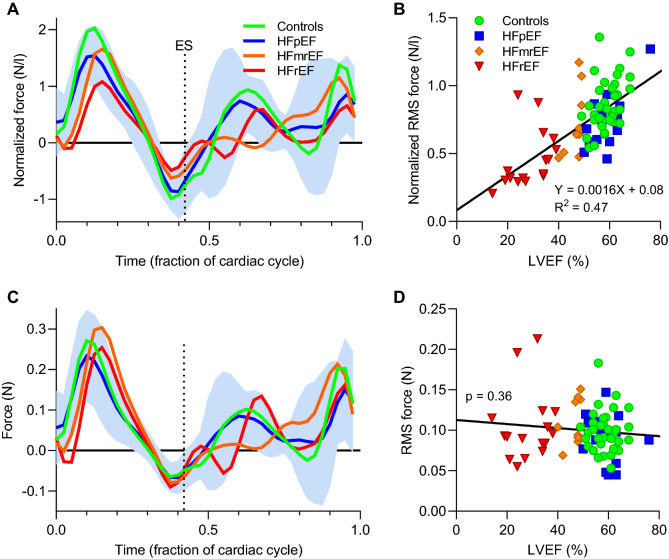
(**A**) Hemodynamic forces in the apical-basal direction, normalized to left ventricular volume, group averages. The light blue shaded area denotes ± 1SD from average values for the HFpEF group. (**B**) Linear regression analysis for EF and volume normalized F_RMS_ for the entire heartbeat demonstrated a strong correlation (p < 0.001). (**C**) Hemodynamic forces without volume normalization, group averages. (**D**) Regression analysis of EF vs F_RMS_ without volume normalization found no significant association.

Average values for longitudinal (apical-basal) forces are shown in Fig. 3. Panel A shows volume-normalized forces for the four groups. Of note, the average values for controls (green line) were found within ± 1SD of the HFpEF average values (blue line and shaded area). A correlation was seen between EF and F_RMS_ for the entire heartbeat (panel B). When volume normalization was not performed, the systolic forces clustered closer together (panel C), and no correlation was seen between EF and non-normalized F_RMS_ for the entire heartbeat (panel D).

**Figure 4:**
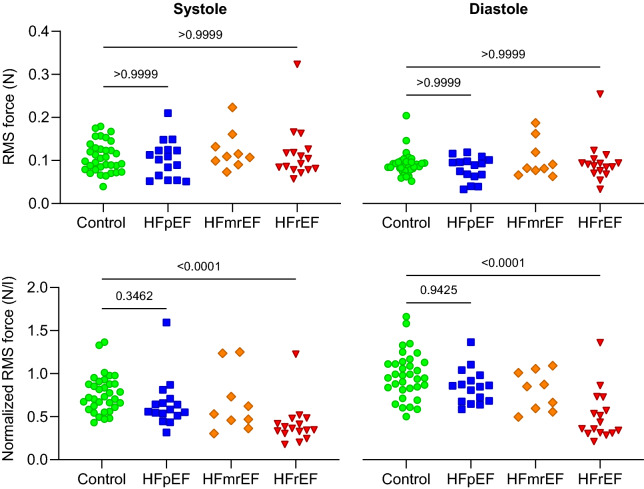
Group comparison of F_RMS_ in systole (left column) and diastole (right), with and without LV volume normalization. Importantly, no significant differences were seen between healthy controls and HFpEF patients.

**Figure 5:**
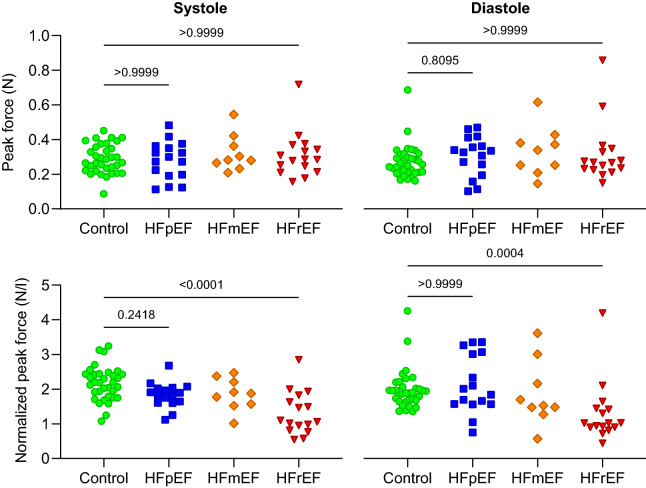
Group comparison of F_Peak_ in systole (left column) and diastole (right), with and without LV volume normalization. Similarly to RMS forces, no differences were seen between controls and HFpEF patients.

Comparing F_RMS_ and F_peak_ values between groups in both systole and diastole, we found no differences between controls and HFpEF patients whether LV volume normalization was performed or not (Figs. 4 and 5). Significant differences were seen between HFrEF and the other groups in volume-normalized data, reflecting the larger ventricular volumes in this patient category.

## Discussion

In this study, we investigated left ventricular hemodynamic forces in healthy subjects and heart failure patients with varying degrees of systolic dysfunction. While some individual HFpEF cases differed appreciably from controls, we found no significant differences on the group level. Although volume-normalized forces correlated with ejection fraction, this correlation was lost when normalization was not performed, indicating LV volume differences rather than hemodynamic mechanisms as the driver of this correlation. From our findings, we conclude that hemodynamic force analysis is currently not ready for clinical application in suspected HFpEF, as it is unable to reliably distinguish between HFpEF and healthy hearts in the resting state.

We found that volume-normalized force in the apical-basal direction correlated with ejection fraction, but importantly the same was not seen for non-normalized force. A trend was seen towards lower forces in the apical-basal direction in patients with impaired ejection fraction, explained by their larger ventricular volumes. Lapinskas et al. previously demonstrated a correlation between EF and volume-normalized force^11^. Their method estimated hemodynamic forces from CMR long-axis images, aortic root dimensions and mitral inflow dimensions, which was previously shown to provide acceptable measures of forces in the apical-basal direction^10^. However, to facilitate comparison between patients with different heart sizes, they also normalized all their data to LV volume. Since end-diastolic volume is inversely related to EF, indexing F_RMS_ to EDV (or time-resolved LV volume) inherently introduces a correlation to EF and will separate groups because of volumetric differences alone when forces are similar. In the present study we examined both normalized and non-normalized forces and found ventricular volume to be the driver of the differences seen. We also did not find any significant differences in forces among controls and patients with normal ejection fraction. Hemodynamic forces are therefore not likely to improve detection of early myocardial systolic dysfunction. Depending on the specific research question, indexing forces to stroke volume or cardiac output may be a better option, since these variables relate more directly to the effective mass transfer of cardiac pumping and hence force output.

Considering the methodology, the approach used herein constitutes the reference standard for obtaining hemodynamic forces. In comparison, the method employed by Lapinskas et al.^11^ computes hemodynamic forces using numerical calculation of mass inflow and outflow from a three-dimensional mesh and known or assumed inflow conditions. In healthy hearts, the majority of ventricular pumping occurs as the result of a longitudinal shortening of the ventricles, known as atrioventricular plane displacement^20^. This mode of contraction is attenuated in acute myocardial injury as well as in heart failure, but remains an important driver of ventricular pumping^21–24^. While the 4D flow method for force quantification is unlikely to be affected by the underlying mechanism of contraction, it is possible that the endocardial dynamics-based model is sensitive to such alterations^10^.

Despite studying a larger population than the previous publication, and using the best currently available method of measurement, we were unable to detect significant differences between HFpEF and controls. The still limited population size may be a contributing factor here, but it is also noteworthy that all examinations were carried out at rest. Furthermore, all HFpEF patients were in a state of compensation during the examination, as indicated by normal oxygen saturation levels, normal blood pressure, and no symptoms. There is gathering evidence for the application of stress testing in suspected heart failure^25,26^. While statistically significant intergroup differences may be found by increasing the study size, it would be of greater clinical utility to use stress imaging to unmask latent differences in cardiac performance. More studies are required to investigate the normal biological inter- and intrasubject variability of hemodynamic forces in different diseases and during altered cardiac loading conditions.

Diastolic function is thought to deteriorate with age^27–29^, and while controls were significantly younger than the aggregated HF group, the difference was driven by the HFpEF patients. This is in keeping with the commonly observed characteristics of HFpEF patients, who are typically older, more frequently diabetic, and less often suffering from ischemic heart disease compared to HFrEF patients^30,31^. Given that diastolic function is the key culprit in HFpEF^25,32,33^, any difference in hemodynamic force between controls and HFpEF patients would therefore be expected to be amplified rather than attenuated by the age difference. Despite this, we saw similar hemodynamic forces in HFpEF and controls, further supporting that force analysis is likely not a robust measure of ventricular function in the absence of decreased ejection fraction.

It is well known that the early diastolic acceleration of blood (E-wave) is caused by ventricular suction^34–36^, manifested as a transient atrioventricular pressure gradient which is affected by alterations in both systolic and diastolic performance^37–39^. Further studies into filling dynamics using hemodynamic force analysis would likely reveal additional information regarding atrioventricular interactions, as the pressure gradient which causes LV filling necessarily involves atrial blood^19^ and blood in the pulmonary veins^40,41^. This gradient has been studied both using invasive measurements^36,37^ and noninvasive 4D flow-derived approaches^42^, but was beyond the scope of the current study.

### Limitations

For our measurements we used a prototype 4D flow sequence. Today, one major MR vendor offers 4D flow as part of the standard clinical package, and the other two are actively supporting research applications. If 4D flow is unavailable, forces can be computed from echo-PIV data^2,43^ as well as from regular cardiovascular magnetic resonance (cine SSFP) images^10^. The SSFP method was compared to reference-standard 4D flow and found to approximate forces in the longitudinal (apex-base) direction (time-averaged RMS R^2^ = 0.77, instantaneous values R^2^ = 0.59) and in the inferolateral-anteroseptal direction to a lesser degree (time-averaged RMS R^2^ = 0.71, instantaneous values R^2^ = 0.25 with larger bias). Of note, the SSFP method performed worse in patients than in healthy volunteers. In contrast, the 4D flow method has been validated against laser particle imaging velocimetry with good accuracy and precision for both peak and RMS forces (R^2^ = 0.96–1.0), high reproducibility and good agreement between scans with and without respiratory gating, for different field strengths, and for different LV segmentation methods^14^.

A remaining issue with 4D flow is the sensitivity to background phase offsets, which we countered by first- or second-order correction. While this is an imperfect solution^16,44–46^, it represents the current state of the art and was performed similarly for all groups, minimizing the risk of intergroup bias in the flow measurements.

A descriptive, cross-sectional study should use the most precise and accurate method of measurement available, to minimize noise and focus on physiological rather than methodological interpretations of data. Therefore, we have used state-of-the-art imaging to demonstrate that a significant part of the previously described intergroup differences arise from the normalization of measured hemodynamic forces to ventricular volumes rather than from underlying differences in ventricular mechanics or other physiological phenomena.

## Conclusions

Left ventricular hemodynamic force analysis, whether indexed to LV volumes or not, fails to distinguish between healthy subjects and patients with heart failure and preserved ejection fraction. While force analysis remains a promising concept for specific applications, the current findings do not support a clear role for hemodynamic forces in HFpEF assessment.

## Data Availability

While the individual CMR datasets supporting the current study are not publicly available due to limitations in our ethical permits, exported/processed data are available from the corresponding author on reasonable request. The analysis software for computation of hemodynamic forces from 4D flow datasets is freely available^14^.

## Abbreviations

CMR: Cardiovascular magnetic resonance
Echo-PIV: Echocardiography with particle imaging velocimetry
F_peak_: Peak hemodynamic force
F_RMS_: Root mean square hemodynamic force
HFpEF: Heart failure with preserved ejection fraction
HFmrEF: Heart failure with mid-range ejection fraction
HFrEF: Heart failure with reduced ejection fraction
LAD: Left anterior descending coronary artery
LGE: Late gadolinium enhancement
RMS: Root mean square
SCAPIS: Swedish CArdioPulmonary bioImage Study
